# Relationship Between Caregiver Burden and Sense of Coherence in Home‐Based Family Caregivers

**DOI:** 10.1155/oti/6638411

**Published:** 2026-01-28

**Authors:** Yasuo Yamamoto

**Affiliations:** ^1^ Department of Rehabilitation, Faculty of Health Sciences, Suzuka University of Medical Science, Suzuka City, Mie Prefecture, Japan, suzuka-u.ac.jp

**Keywords:** caregivers, cross-sectional studies, occupational therapy, sense of coherence

## Abstract

**Objectives:**

Family caregivers often face significant challenges, such as insufficient sleep, fatigue due to aging, difficulty engaging in social activities, and fewer interactions with friends and neighbors. However, some family caregivers manage to maintain active lifestyles while providing home care. This study is aimed at exploring the relationship between family caregivers′ sense of coherence (SOC) and their perception of caregiving burden.

**Methods:**

This cross‐sectional study recruited 74 family caregivers of community‐dwelling older adults who used day‐care insurance services; 64 were included in the final analysis. Data were collected using a questionnaire that included the SOC‐13 scale, rated on a 5‐point Likert scale. Multiple regression analysis was conducted to identify the factors influencing caregivers′ perception of caregiving burden, with SOC subfactors serving as explanatory variables.

**Results:**

Comprehensibility, one of the SOC subscales, showed a significant negative association with caregiver burden, and the association was strengthened after adjustment (Model 2: *B* = −0.376, *β* = −0.534, *p* = 0.005). Daily caregiving hours were positively associated with burden, whereas meaningfulness and manageability were not significant.

**Conclusions:**

This study highlights the importance of comprehensibility in influencing caregivers′ sense of burden. The findings provide valuable insights into the relationship between caregivers′ SOC and their perceived caregiving burden. Moreover, they emphasize the relevance of this relationship to the field of occupational therapy, as occupational therapists can play a key role in enhancing caregivers′ comprehensibility, thereby helping them alleviate the caregiving burden through targeted interventions.

**Clinical Implications:**

These findings underscore the importance of promoting comprehensibility among family caregivers to alleviate their perceived burden and support sustained caregiving. Occupational therapy interventions focused on improving caregivers′ understanding of the caregiving process can contribute to better mental and physical health outcomes, benefiting both caregivers and care recipients.

## 1. Introduction

Although the rate of increase varies by country, the global proportion of the older‐adult population is on the rise [1]. In Japan, the number of people requiring care is increasing due to the aging population; thus there is also an increase in the number of family caregivers. Additionally, the burden per family caregiver is expected to increase in the future because of the declining population and shrinking family size [1]. In such a society, junior baby boomers, who are busy with work and child‐rearing, are expected to take on the responsibility of caring for their parents; thus, home‐based family caregiving has a significant impact on society. Family caregivers spend more than half a day in caregiving [2]; have shorter sleep time and lower sleep quality [3]; cannot frequently participate in social activities [4]; and feel more isolated, with fewer interactions with friends and neighbors [5, 6]. Family caregivers′ sense of caregiving burden is related to the physical and functional levels of the person requiring care [7, 8] and the degree of caregiving burden is related to the risk of abuse [9].

On the other hand, there are family caregivers who, while providing care, manage to live a life that aligns with their own values. In other words, family caregivers who provide care while living their lives have learned to recognize their circumstances and manage them effectively, thereby avoiding the burden. This can be viewed as an ability to cope with stress, known as sense of coherence (SOC), which has been receiving increasing attention. SOC is a central concept in salutogenesis and represents the ability to maintain and enhance health even under difficult circumstances. This concept is closely related to the practical approaches of occupational therapy where the focus is on utilizing an individual′s strengths and resources within their life to support a healthy and meaningful life, which aligns closely with the concept of SOC. Even under stress, people possess psychological and cognitive mechanisms that enable them to generate their own health and adapt to situations, and SOC plays a role in this process [10]. In occupational therapy, by utilizing SOC, caregivers can maintain their health and adapt to the burden of caregiving. Promoting the health of caregivers not only improves their own quality of life (QOL) but also positively affects the QOL of those in need of care. By maintaining psychological and physical health, caregivers are able to provide more stable, high‐quality care, ultimately benefiting the care recipients.

SOC consists of three components: “comprehensibility,” which is the belief that environmental stimuli can be predicted and explained in an orderly manner; “manageability,” which is the sense that resources to cope with these demands are always available; and “meaningfulness,” which is the belief that the demands are challenges worth engaging in with full effort [10]. Later, based on empirical research, Yamazaki defined SOC as “the ability or source that enables individuals to protect their mental and physical health by effectively mobilizing their internal and external resources in response to stressful events and situations, and to live a bright and fulfilling life, turning these experiences into growth and development” [11].

Previous studies have reported various factors that reduce caregiver burden, such as the care recipient′s basic functional abilities and daily living skills, and the availability of someone to assist or consult with the primary caregiver [12], and reducing the caregiver′s physical and mental fatigue [13]. Regarding the relationship between caregiver SOC and caregiver burden, studies have shown that SOC affects emotional burden and depression levels [14] and that higher SOC reduces caregiver burden and improves QOL [15, 16]. However, it remains unclear which component of SOC (“comprehensibility,” “manageability,” or “meaningfulness”) influences caregiver burden and in what way, which is an important issue to be clarified in future research.

In this study, we are aimed at clarifying the relationship between SOC and caregiver burden by integrating the perspective of SOC. By examining how the three components of SOC influence the caregiver burden of family caregivers living with care recipients at home, we are aimed at demonstrating how occupational therapy practices contribute to the health maintenance and promotion of family caregivers. Additionally, it is expected that these findings will play a significant role in supporting the continued community life of older individuals who require assistance or long‐term care.

## 2. Materials and Methods

### 2.1. Research Participants

**Figure 1:**
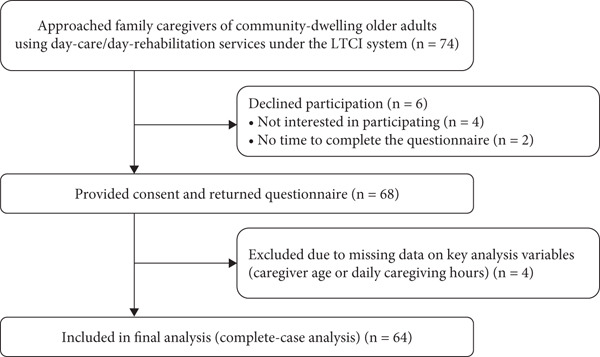
Flow diagram of participant recruitment and inclusion.

We targeted family caregivers of 74 community‐dwelling older adults who had been certified as requiring long‐term care and were using day‐care rehabilitation or day‐care services under the long‐term care insurance (LTCI) system. Six caregivers declined to participate: four were not interested in participating and two reported that they did not have enough time to complete the questionnaire. Therefore, 68 caregivers who were invited to participate and provided informed consent were included in the study. Of the 68 caregivers, four were excluded due to missing data on key analysis variables (caregiver age or daily caregiving hours), leaving 64 caregivers for the final analysis. The overall selection process is summarized in Figure 1.

This study was approved by the Suzuka University Medical Science Ethics Committee (Approval Number 542: July 4, 2023). Data were collected between June 1 and November 30, 2023.

This study followed the Strengthening the Reporting of Observational Studies in Epidemiology (STROBE) guidelines for reporting cross‐sectional studies [17, 18].

### 2.2. Survey Method

The survey method consisted of a 15–20‐min questionnaire for each family caregiver and an SOC‐13 item 5‐point rated scale, administered once using the retention method. The questionnaire consisted of basic information, such as age, sex, and level of care required by the patient; the age and sex of family caregivers; and daily caregiving hours and sense of caregiver burden. The specifications are described below.

1. The care level of recipients was determined by the LTCI system in Japan. Japan′s LTCI system was introduced in the year 2000 to address the needs of an aging society. The system classifies individuals into seven levels based on physical and cognitive disabilities: Those requiring assistance are classified as “Support 1–2” and those requiring nursing care as “Nursing Care 1–5.”
2. Daily caregiving hours

Although it is difficult to completely distinguish between caregiving time and personal time, we quoted Yamada et al.: “time that caregivers themselves perceive as being spent on caregiving” [19] and obtained responses regarding the average daily caregiving time over 3 weeks, as subjectively perceived.

3. Caregiver burden

The visual analogue scale (VAS) [20] was used to assess the caregiver burden. This scale was used because it is intended for home‐based family caregivers with a risk of mental stress. Further, it is a short and efficient measurement, which reduces the burden of responses. The VAS has been validated as a means of measuring stress [21]. Caregiver burden was assessed on a 10‐point numeric scale anchored at “not at all burdensome” (0) and *“*very burdensome” (10). Participants indicated their perceived burden, and the score (0–10) was used in the analyses; higher scores indicate greater burden.

4. SOC

For SOC, we adopted the definition by Yamazaki [11]. SOC is considered measurable, and there is a 29‐item, 13‐item, and 3‐item version of the SOC scale. Of these, the 29‐ and 13‐item versions have been validated for internal consistency, reliability, and validity [10]. Given that the reliability of the SOC‐13 scale has been supported by previous studies, we used the 13‐item SOC scale rated on a 5‐point Likert scale to reduce the response burden. In addition, we examined the internal consistency of the Japanese version of the SOC‐13 using Cronbach′s *α* coefficients for both the total scale and the three subscales. The SOC‐13 consists of four “meaningfulness” items, five “comprehensibility” items, and four “manageability” items. For each question, a self‐assessment was made using a 5‐point scale ranging from “not at all (1 point)” to “very often (5 points).” All items were summed to obtain a score, with a higher score indicating a higher SOC [10].

### 2.3. Analysis Method

#### 2.3.1. Target Audience Attributes and SOC Scores

To confirm the collective characteristics of the participants, we conducted descriptive statistics for the basic attributes of care recipients and family caregivers, the daily care hours, caregiver burden, and the total score of SOC and its subitems (meaningfulness, comprehensibility, and manageability). Further, we examined the internal consistency of the Japanese version of the SOC‐13 using Cronbach′s *α* coefficients for both the total scale and the three subscales.

#### 2.3.2. Association Between SOC of Family Caregivers and Their Sense of Caregiving Burden: A Single Regression Analysis

To clarify the relationship between SOC and caregiver burden among family caregivers, we conducted a single regression analysis using caregiver burden as the objective variable and SOC total score and its subitems as explanatory variables.

#### 2.3.3. Associations Between Family Caregivers′ SOC Subfactor Scores (Meaningfulness, Comprehensibility, and Manageability) and Sense of Care Burden: A Multiple Regression Analyses

To clarify the relationship between the three SOC subitems and caregiver burden, a multiple regression analysis was conducted using caregiver burden as the objective variable and meaningfulness, comprehensibility, and manageability as explanatory variables, adjusting for age, sex, care level, and daily care hours of family caregivers. In addition, we performed correlation analyses among the study variables, and the results are presented in Supporting Information 2 (Table S1). *p* < 0.05 was considered significant for all tests. Statistical analyses were performed using the SPSS Statistics Version 29.0. In addition, a sensitivity power analysis was conducted using G∗Power (Version 3.1). With *n* = 64, *α* = 0.05, and three predictors (SOC subscales), the calculated effect size threshold was *f* ^2^ = 0.286, indicating sufficient power (> 0.80) to detect medium‐to‐large effects. When including covariates (seven predictors), the threshold increased to *f* ^2^ = 0.386, suggesting that the study was adequately powered to detect only large effects.

## 3. Results and Discussion

### 3.1. Characteristics of the Target Population (Care Recipients and Family Caregivers) (Table 1)

**Table 1:** Attributes of care recipients and family caregivers and their sense of coherence.

	**Mean (SD)**
Care recipients	
Age, years	83.3 (7.2)
Sex, *n* (%)	
Male	31 (48.4)
Female	33 (51.6)
LTCI (care level), *n* (%)^a^	
Support Level 1	11 (17.2)
Support Level 2	11 (17.2)
Care Level 1	10 (15.6)
Care Level 2	13 (20.3)
Care Level 3	5 (7.8)
Care Level 4	7 (10.9)
Care Level 5	7 (10.9)
Family caregiver	
Age, years	68.9 (10.7)
Sex, *n* (%)	
Male	17 (26.6)
Female	47 (73.4)
Daily caregiving hours (h)	9.4 (7.9)
Caregiver burden^b^	5.0 (2.8)
SOC‐13 score^c^	
Total	45.1 (9.1)
Meaningfulness	14.3 (2.5)
Comprehensibility	17.1 (3.9)
Manageability	13.8 (3.7)

*Note:* Age, daily caregiving hours, caregiver burden, and SOC are means (SD). Complete‐case (listwise) analysis; *n* = 64.

Abbreviations: LTCI, long‐term care insurance; SD, standard deviation; SOC, sense of coherence; VAS, visual analogue scale.

^a^ LTCI (care level): support levels (1–2); care levels (1–5).

^b^ Caregiver burden: VAS (0–10).

^c^ SOC‐13: 13‐item scale rated on a 5‐point scale. Total score (13–65); meaningfulness (4–20); comprehensibility (5–25); manageability (4–20).

Of the 68 returned questionnaires, four were excluded due to missing data on key analysis variables (two missing caregiver age and two missing daily caregiving hours). We used listwise deletion; therefore, cases with missing data on any key variable were excluded. As a result, data from 64 participants were included in the final analysis (care recipients: 31 males and 33 females; family caregivers: 17 males and 47 females). Thus, we conducted a complete‐case analysis (listwise deletion), including only participants with complete data on all key variables. The mean age of care recipients was 83.3 years (SD 7.2). Family caregivers had a mean age of 68.9 years (SD 10.7) and reported average daily caregiving hours of 9.4 h (SD 7.9). The mean caregiver‐burden score (VAS 0–10) was 5.0 (SD 2.8). The mean total SOC‐13 score was 45.1 (SD 9.1), with subscale means of 14.3 (SD 2.5) for meaningfulness, 17.1 (SD 3.9) for comprehensibility, and 13.8 (SD 3.7) for manageability. The Cronbach′s *α* coefficients in this study were 0.79 for the total SOC‐13, 0.41 for meaningfulness, 0.41 for comprehensibility, and 0.51 for manageability, indicating good internal consistency for the total scale and modest internal consistency for the subscales. Family caregivers were predominantly female (73.4%). Among care recipients, the most frequent category under the LTCI system was Care Level 2 (20.3%).

### 3.2. Association Between Family Caregiver Burden and SOC (Including Subfactors): A Single‐Linear Analysis (Table 2)

**Table 2:** Association between family caregiver burden and SOC.

	**Simple linear analysis**			**Multiple regression analysis**							
				**Model 1**				**Model 2**			
SOC	*B*	*p* value	95% CI	*B*	*β*	*p* value	95% CI	*B*	*β*	*p* value	95% CI
Total	−0.165	< 0.001 ^∗∗∗^	−0.230, −0.100								
Meaningfulness	−0.427	0.002 ^∗∗^	−0.684, −0.170	−0.091	−0.086	0.611	−0.448, 0.266	0.021	0.020	0.895	−0.295, 0.337
Comprehensibility	−0.394	< 0.001 ^∗∗∗^	−0.548, −0.240	−0.321	−0.457	0.032 ^∗^	−0.613, −0.029	−0.376	−0.534	0.005 ^∗∗^	−0.632, −0.120
Manageability	−0.392	< 0.001 ^∗∗∗^	−0.559, ‐0.225	−0.020	−0.026	0.915	−0.386, 0.347	0.009	0.012	0.953	−0.311, 0.330

*Note:* *B*, unstandardized regression coefficient; *β*, standardized regression coefficient. Model 1 is a multiple regression analysis in which meaningfulness, comprehensibility, and manageability are simultaneously included. Model 2 is a multiple regression analysis in which covariates such as caregiver age, sex, daily caregiving hours, and LTCI were added to Model 1. Complete‐case (listwise) analysis; *n* = 64.

Abbreviations: 95% CI, 95% confidence interval; CI, confidence interval; SOC, sense of coherence; LTCI, long‐term care insurance.

^∗^ *p* < 0.05.

^∗∗^ *p* < 0.01.

^∗∗∗^ *p* < 0.001.

A single‐linear analysis was conducted using caregiver burden as the objective variable and the total SOC score and its subitems as explanatory variables. The results showed that the total SOC score (*p* < 0.001), sense of meaningfulness (*p* = 0.002), comprehensibility (*p* < 0.001), and manageability (*p* < 0.001) were all related.

### 3.3. Associations Between Family Caregivers′ SOC Subfactors (Meaningfulness, Comprehensibility, and Manageability) and Caregiver Burden: A Multiple Regression Analysis (Table 2)

Model 1 (unadjusted): Caregiver burden (VAS) was considered the dependent variable, and the three SOC subscales—comprehensibility, manageability, and meaningfulness—were the predictors. Only comprehensibility showed a significant negative association with caregiver burden (*B* = −0.321, *β* = −0.457, *p* = 0.032, 95% CI [−0.613, −0.029]), whereas manageability and meaningfulness were not significant.

Model 2 (adjusted): After adjusting for caregiver age, sex, daily caregiving hours, and LTCI level, the negative association for comprehensibility became stronger (*B* = −0.376, *β* = −0.534, *p* = 0.005, 95% CI [−0.632, −0.120]). Daily caregiving hours were positively associated with burden (*B* = 0.136, *p* < 0.001). Other covariates were not significant (LTCI was borderline, *p* ≈ 0.050). No problematic multicollinearity was observed (maximum VIF < 5). See Table 2 for full coefficients and 95% CIs.

### 3.4. Discussion

This study examined the relationship between caregiver burden and SOC in family caregivers.

All care recipients in this study were certified under Japan′s LTCI system. As shown in Table 1, their care levels ranged from 1 to 5, indicating heterogeneous care needs. Accordingly, the present findings are primarily applicable to family caregivers of older adults who use day‐care insurance services and should be generalized to caregivers of home‐visit service users (e.g., home‐visit nursing or rehabilitation) with caution.

#### 3.4.1. Association Between Family Caregiver Burden and SOC (Table 2)

The results of the simple linear regression indicated that caregiver burden was negatively associated with all SOC measures: SOC‐13 total (*p* < 0.001), meaningfulness (*p* = 0.002), comprehensibility (*p* < 0.001), and manageability (*p* < 0.001).

In the multiple linear regression, comprehensibility showed a significant negative association with caregiver burden in both models and the association became stronger after adjustment. Specifically, Model 1 (unadjusted): *B* = −0.321, *β* = −0.457, *p* = 0.032, 95% CI [−0.613, −0.029]; Model 2 (adjusted for caregiver age, sex, daily caregiving hours, and LTCI level): *B* = −0.376, *β* = −0.534, *p* = 0.005, 95% CI [−0.632, −0.120]. Daily caregiving hours were positively associated with burden (*B* = 0.136, *p* < 0.001), whereas meaningfulness and manageability were not significant. In multiple regression, the standardized regression coefficient (*β*) reflects relative influence in the model; in Model 2, the coefficients were meaningfulness (*β* = 0.068), comprehensibility (*β* = −0.534), and manageability (*β* = −0.109). The standardized *β* coefficient for comprehensibility (*β* = −0.534) indicates a moderate‐to‐strong negative association with caregiver burden. Practically, this suggests that caregivers who can better understand and make sense of their caregiving situations tend to perceive their caregiving tasks as less burdensome.

Comprehensibility refers to a sense of confidence that stimuli arising from one′s internal and external environment are structured, predictable, and explicable. In this study, comprehensibility was more strongly related to caregiver burden than meaningfulness or manageability, suggesting that in the highly uncertain context of family caregiving, the ability to understand what is happening and to anticipate future developments may reduce psychological stress.

In actual caregiving situations, caregivers frequently face sudden behaviors and must assist with daily activities while responding to changes in cognitive and physical function. The ability to accurately appraise questions such as “Why is this occurring now?” or “What response is needed?” may help caregivers perceive care as manageable and reduce anxiety and stress. Because comprehensibility is shaped by knowledge, experience, and access to supportive resources, interventions such as information provision, disease‐specific education programs, the use of assessment tools and care manuals, and regular communication with care managers may be effective. Psychosocial support for dementia caregivers should also consider caregiver characteristics. To further clarify these associations, we examined intervariable correlations (see Supporting Information 2, Table S1). Comprehensibility showed a weak, nonsignificant negative correlation with daily caregiving hours (*r* = −0.057, *p* > 0.05), whereas caregiver burden correlated positively with caregiving hours (*r* = 0.389, *p* < 0.01) and negatively with comprehensibility (*r* = −0.535, *p* < 0.01). Taken together, these findings suggest that adjustment for daily caregiving hours clarified and strengthened the independent association of comprehensibility with caregiver burden in Model 2.

#### 3.4.2. Significance and Limitations of the Study

This finding suggests that, in complex and stressful caregiving contexts, whether caregivers can perceive the situations they face as understandable and predictable has a meaningful impact on their subjective burden. This provides an important implication for caregiver support in occupational therapy: Support should not be limited to physical assistance but should actively address psychosocial aspects. From an occupational therapy perspective, our results highlight that enhancing caregivers′ “comprehensibility”—their sense that internal and external stimuli are structured, predictable, and explicable—may be particularly helpful in reducing psychological burden.

However, because this was a cross‐sectional study, causal inference is limited. The association between comprehensibility and caregiver burden may be bidirectional, and this possibility should be considered; longitudinal follow‐up studies are needed to clarify temporal ordering and causality.

In the multiple regression analysis, we adjusted for a limited set of covariates (caregiver age, sex, care‐dependency level, and daily caregiving hours), whereas other psychosocial factors (e.g., living arrangement, employment status, depressive symptoms, and social support) were not included. Therefore, residual confounding cannot be ruled out, and the findings should be interpreted with caution. In addition, participants were family caregivers of older adults using day‐care/day‐rehabilitation services, so generalizability to caregivers who do not use such services may be limited. Further, we used complete‐case (listwise) deletion. If missingness was not completely random, selection bias may have occurred. The proportion excluded due to missingness was small (4/68; ≈5.9*%*); thus, the impact is likely limited; nevertheless, future research should consider approaches such as multiple imputation to reduce potential bias.

Another methodological limitation concerns the internal consistency of the SOC‐13 subscales in this study, which was modest (*α* = 0.41–0.51). These relatively low *α* values may reflect not only measurement error but also the small number of items included in each subscale and the conceptual breadth covered by each dimension of SOC. From a psychometric perspective, lower internal consistency generally biases regression coefficients toward the null, meaning that any true associations are more likely to be underestimated than exaggerated. Therefore, the significant association observed between comprehensibility and caregiver burden in our multiple regression model is likely to represent a conservative estimate of the true relationship. Nevertheless, the modest reliability of the SOC‐13 subscales should be regarded as a limitation of this study, and future research using scales with higher internal consistency or alternative measures of SOC is warranted.

In light of these limitations, the present findings emphasize the importance of targeting psychological/cognitive components in caregiver support. Interventions that strengthen comprehensibility—for example, structured information provision, disease‐specific education, practical assessment tools and care manuals, and regular communication channels with care managers—may help caregivers make sense of care demands and reduce stress. These insights can inform the development and testing of future caregiver‐support programs. In addition, a sensitivity power analysis indicated that our study had sufficient power to detect medium‐to‐large effects (*f* ^2^ = 0.286) when focusing on SOC subscales; however, only large effects (*f* ^2^ = 0.386) could be reliably detected when covariates were included. Therefore, smaller effects may not have been captured, and future studies with larger samples are warranted.

## 4. Conclusion

This study is aimed at identifying factors associated with caregiving burden from the perspective of SOC, focusing on family caregivers of users of day‐care rehabilitation and day‐care services. The analysis revealed that among the subcomponents of SOC, “comprehensibility” was significantly associated with caregiving burden. These findings suggest that whether caregivers perceive their caregiving situation as “understandable and predictable” has a substantial impact on their subjective burden. Specifically, enhancing caregivers′ “comprehensibility” may be an effective psychological intervention to alleviate caregiving burden.

The results highlight the importance of focusing on psychological factors in caregiver support. This study emphasizes that occupational therapists should not only provide physical support but also actively intervene in the psychosocial aspects of caregiving. Future research should examine specific methods of support and interventions aimed at increasing caregivers′ comprehensibility through longitudinal and comprehensive empirical studies.

## Conflicts of Interest

The author declares no conflicts of interest.

## Funding

No funding was received for this manuscript.

## Supporting Information

Additional supporting information can be found online in the Supporting Information section.

## Supporting information


**Supporting Information 1** File S1: STROBE checklist for cross‐sectional study.

**Supporting Information 2** Table S1: Correlation matrix among study variables.

## Data Availability

The data that support the findings of this study are available from the corresponding author upon reasonable request.
