# Supplementary material for: Relationship Between Caregiver Burden and Sense of Coherence in Home‐Based Family Caregivers
Source: Occup Ther Int. 2026 Jan 28;2026:6638411. doi: 10.1155/oti/6638411 (PMC12848607; doi:10.1155/oti/6638411)
Supplement: Supplementary file 1 — Supporting Information 1 File S1: STROBE checklist for cross‐sectional study. [file OTI-2026-6638411-s001.docx]

The STROBE reporting checklist

For checking that observational epidemiology research articles can be understood and used by everyone

| 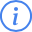 Note |
| --- |
| If you have not used a reporting guideline before, read about [how and why to use them](https:/resources.equator-network.org/about/reporting-guidelines.html) and check whether STROBE is the [most applicable reporting guideline](https:/resources.equator-network.org/reporting-guidelines/strobe/index.html?#applicability) for your work.  Reporting guidelines are most useful when used early in research. When writing a manuscript or application, consider using the [Full Guidance](https:/resources.equator-network.org/reporting-guidelines/strobe/index.html) where you’ll see explanations and examples for each item.  After writing, demonstrate adherence by completing this checklist:   1. Specify where each item is described (see [Note 1](#sec-specify)). 2. Cite this checklist (See [Note 2](#sec-cite)). 3. Include your completed checklist as a supplement when submitting to a journal so that future readers can use it to find information. |

|  | Item Description | Location (or reason for not reporting) |
| --- | --- | --- |
| **Title and abstract** |  |  |
| [1a. Indicate the study’s design](https:/resources.equator-network.org/reporting-guidelines/strobe/items/title-abstract-indicate-study-design.html?utm_source=strobe&utm_medium=checklist&utm_campaign=1_1) | Indicate the study’s design with a commonly used term in the title or the abstract. | Abstract; Materials and methods – 2.1 Research Participants (cross-sectional questionnaire survey). |
| [1b. Abstract](https:/resources.equator-network.org/reporting-guidelines/strobe/items/abstract.html?utm_source=strobe&utm_medium=checklist&utm_campaign=1_1) | Provide in the abstract an informative and balanced summary of what was done and what was found. | Abstract (Objectives, Methods, Results, and Conclusions). |
| **Introduction** |  |  |
| [2. Background / rationale](https:/resources.equator-network.org/reporting-guidelines/strobe/items/background-rationale.html?utm_source=strobe&utm_medium=checklist&utm_campaign=1_1) | Explain the scientific background and rationale for the investigation being reported. | Introduction (background on caregiver burden, SOC, and occupational therapy; first to fourth paragraphs). |
| [3. Objectives](https:/resources.equator-network.org/reporting-guidelines/strobe/items/objectives.html?utm_source=strobe&utm_medium=checklist&utm_campaign=1_1) | State specific objectives, including any prespecified hypotheses. | Introduction (final paragraph stating the aim to clarify the relationship between SOC and caregiver burden). |
| **Methods** |  |  |
| [4. Study design](https:/resources.equator-network.org/reporting-guidelines/strobe/items/study-design.html?utm_source=strobe&utm_medium=checklist&utm_campaign=1_1) | Present key elements of study design early in the paper. | Materials and methods – 2.1 Research Participants; 2.2 Survey method; 2.3 Analysis Method (cross-sectional questionnaire survey and regression analyses). |
| [5. Setting](https:/resources.equator-network.org/reporting-guidelines/strobe/items/setting.html?utm_source=strobe&utm_medium=checklist&utm_campaign=1_1) | Describe the setting, locations, and relevant dates, including periods of recruitment, exposure, follow-up, and data collection. | Materials and methods – 2.1 Research Participants (family caregivers of community-dwelling older adults using day-care/day-rehabilitation services in Japan). Recruitment and data collection were conducted between June 1 and November 30, 2023. |
| [6a. Eligibility criteria](https:/resources.equator-network.org/reporting-guidelines/strobe/items/eligibility-criteria.html?utm_source=strobe&utm_medium=checklist&utm_campaign=1_1) | **Cohort study:** Give the eligibility criteria, and the sources and methods of selection of participants. Describe methods of follow-up. **Case-control study:** Give the eligibility criteria, and the sources and methods of case ascertainment and control selection. Give the rationale for the choice of cases and controls. **Cross-sectional study:** Give the eligibility criteria, and the sources and methods of selection of participants. | Materials and methods – 2.1 Research Participants (eligibility and selection of family caregivers: 74 approached, 68 consented, 64 included in analyses). |
| [6b. Matching criteria](https:/resources.equator-network.org/reporting-guidelines/strobe/items/matching-criteria.html?utm_source=strobe&utm_medium=checklist&utm_campaign=1_1) | **Cohort study:** For matched studies, give matching criteria and number of exposed and unexposed. **Case-control study:** For matched studies, give matching criteria and the number of controls per case. | Not applicable – no matching was used; cross-sectional study. |
| [7. Variables](https:/resources.equator-network.org/reporting-guidelines/strobe/items/variables.html?utm_source=strobe&utm_medium=checklist&utm_campaign=1_1) | Clearly define all outcomes, exposures, predictors, potential confounders, and effect modifiers. Give diagnostic criteria, if applicable. | Materials and methods – 2.2 Survey method (definitions and scoring of caregiver burden VAS, SOC-13 total and subscales, LTCI care level, and daily caregiving hours); 2.3.2 and 2.3.3 (specification of dependent and independent variables in regression models). |
| [8. Data sources / measurement](https:/resources.equator-network.org/reporting-guidelines/strobe/items/data-sources-measurement.html?utm_source=strobe&utm_medium=checklist&utm_campaign=1_1) | For each variable of interest give sources of data and details of methods of assessment (measurement). Describe comparability of assessment methods if there is more than one group. | Materials and methods – 2.2 Survey method (sources of data and measurement: LTCI certification records and caregiver self-administered questionnaire, SOC-13 scale). |
| [9. Bias](https:/resources.equator-network.org/reporting-guidelines/strobe/items/bias.html?utm_source=strobe&utm_medium=checklist&utm_campaign=1_1) | Describe any efforts to address potential sources of bias. | Materials and methods – 2.1 Research Participants (all eligible family caregivers at participating facilities were invited); Discussion – 3.4.3 Significance and Limitations of the Study (selection bias, limited covariates, and residual confounding). |
| [10. Study size](https:/resources.equator-network.org/reporting-guidelines/strobe/items/study-size.html?utm_source=strobe&utm_medium=checklist&utm_campaign=1_1) | Explain how the study size was arrived at. | Materials and methods – 2.3 Analysis Method (description of sample size and regression models); Results – 3.1 and Discussion – 3.4.3 (comment on small sample and detectable effect sizes). |
| [11. Quantitative variables](https:/resources.equator-network.org/reporting-guidelines/strobe/items/quantitative-variables.html?utm_source=strobe&utm_medium=checklist&utm_campaign=1_1) | Explain how quantitative variables were handled in the analyses. If applicable, describe which groupings were chosen, and why. | Materials and methods – 2.2 Survey method (continuous treatment of SOC scores and caregiver burden VAS); 2.3.2 and 2.3.3 (use of continuous explanatory and outcome variables in linear regression; LTCI care level treated as categorical). |
| [12a. Statistical methods](https:/resources.equator-network.org/reporting-guidelines/strobe/items/statistical-methods-description.html?utm_source=strobe&utm_medium=checklist&utm_campaign=1_1) | Describe all statistical methods, including those used to control for confounding. | Materials and methods – 2.3 Analysis Method; 2.3.1–2.3.3 (statistical methods including correlation analysis, single and multiple linear regression, adjustment for caregiver age, sex, daily caregiving hours, and LTCI level; use of SPSS Statistics; significance level p < 0.05). |
| [12b. Statistical methods – subgroups and interactions](https:/resources.equator-network.org/reporting-guidelines/strobe/items/statistical-methods-subgroups-interactions.html?utm_source=strobe&utm_medium=checklist&utm_campaign=1_1) | Describe any methods used to examine subgroups and interactions. | Not applicable – no formal subgroup or interaction analyses were conducted. |
| [12c. Statistical methods – missing data](https:/resources.equator-network.org/reporting-guidelines/strobe/items/statistical-methods-missing-data.html?utm_source=strobe&utm_medium=checklist&utm_campaign=1_1) | Explain how missing data were addressed. | Materials and methods – 2.1 Research Participants (4 caregivers with missing age or daily caregiving hours excluded; complete-case [listwise] analysis); Results – 3.1 (final sample n = 64); Discussion – 3.4.3 (possible selection bias due to listwise deletion). |
| [12di. Statistical methods – loss to follow-up](https:/resources.equator-network.org/reporting-guidelines/strobe/items/statistical-methods-loss-to-follow-up.html?utm_source=strobe&utm_medium=checklist&utm_campaign=1_1) | **Cohort study:** If applicable, describe how loss to follow-up was addressed. | Not applicable – cross-sectional study without follow-up. |
| [12dii. Statistical methods – matching cases and controls](https:/resources.equator-network.org/reporting-guidelines/strobe/items/statistical-methods-matching-cases-controls.html?utm_source=strobe&utm_medium=checklist&utm_campaign=1_1) | **Case-control study:** If applicable, explain how matching of cases and controls was addressed. | Not applicable – no case–control matching design was used. |
| [12diii. Statistical methods – sampling strategy](https:/resources.equator-network.org/reporting-guidelines/strobe/items/statistical-methods-analytical-methods-sampling-strategy.html?utm_source=strobe&utm_medium=checklist&utm_campaign=1_1) | **Cross-sectional study:** If applicable, describe analytical methods taking account of sampling strategy. | Materials and methods – 2.1 Research Participants (all eligible family caregivers at participating facilities were approached; simple recruitment without complex sampling); no special analytical methods for sampling strategy were required. |
| [12e. Statistical methods – sensitivity analyses](https:/resources.equator-network.org/reporting-guidelines/strobe/items/statistical-methods-sensitivity-analyses.html?utm_source=strobe&utm_medium=checklist&utm_campaign=1_1) | Describe any sensitivity analyses. | Not applicable – no sensitivity analyses of the regression results were conducted. |
| **Results** |  |  |
| [13a. Participant numbers](https:/resources.equator-network.org/reporting-guidelines/strobe/items/participants-numbers.html?utm_source=strobe&utm_medium=checklist&utm_campaign=1_1) | Report the numbers of individuals at each stage of the study—e.g., numbers potentially eligible, examined for eligibility, confirmed eligible, included in the study, completing follow-up, and analysed; Consider use of a flow diagram. | Materials and methods – 2.1 Research Participants (74 approached; 68 consented; 64 included in analyses); Results – 3.1 Characteristics of the target population (care recipients and family caregivers). |
| [13b. Participants – non-participation](https:/resources.equator-network.org/reporting-guidelines/strobe/items/participants-non-participation.html?utm_source=strobe&utm_medium=checklist&utm_campaign=1_1) | Give reasons for non-participation at each stage. | Materials and methods – 2.1 Research Participants (four caregivers not interested in participating; two too busy to complete the questionnaire; four additional cases excluded due to missing key variables). |
| [13c. Participants – flow diagram](https:/resources.equator-network.org/reporting-guidelines/strobe/items/participants-flow-diagram.html?utm_source=strobe&utm_medium=checklist&utm_campaign=1_1) | Consider use of a flow diagram. | Methods – 2.1 Research Participants; Figure 1. Participant flow diagram (74 approached, 68 consented, 64 included in analyses). |
| [14a. Descriptive data – participant characteristics](https:/resources.equator-network.org/reporting-guidelines/strobe/items/descriptive-data-participant-characteristics.html?utm_source=strobe&utm_medium=checklist&utm_campaign=1_1) | Give characteristics of study participants (e.g., demographic, clinical, social) and information on exposures and potential confounders. Present the information in a table. | Results – 3.1 Characteristics of the target population (care recipients and family caregivers); Table 1 (demographic, clinical, and caregiving characteristics, SOC scores, and caregiver burden VAS). |
| [14b. Descriptive data – missing data](https:/resources.equator-network.org/reporting-guidelines/strobe/items/descriptive-data-missing-data.html?utm_source=strobe&utm_medium=checklist&utm_campaign=1_1) | Indicate the number of participants with missing data for each variable of interest. | Materials and methods – 2.1 Research Participants; Results – 3.1 (4 cases excluded due to missing age or daily caregiving hours; analyses based on n = 64). Numbers of missing data for each variable are not separately reported. |
| [14c. Descriptive data – follow-up time](https:/resources.equator-network.org/reporting-guidelines/strobe/items/descriptive-data-follow-up-time.html?utm_source=strobe&utm_medium=checklist&utm_campaign=1_1) | **Cohort study:** Summarise follow-up time—e.g., average and total amount. | Not applicable – cross-sectional study without follow-up time. |
| [15. Outcome data](https:/resources.equator-network.org/reporting-guidelines/strobe/items/outcome-data.html?utm_source=strobe&utm_medium=checklist&utm_campaign=1_1) | **Cohort study:** Report numbers of outcome events or summary measures over time. **Case-control study:** Report numbers in each exposure category, or summary measures of exposure. **Cross-sectional study:** Report numbers of outcome events or summary measures. | Results – 3.1 and Table 1 (summary of caregiver burden VAS scores and SOC measures as continuous outcomes). |
| [16a. Main results](https:/resources.equator-network.org/reporting-guidelines/strobe/items/main-results.html?utm_source=strobe&utm_medium=checklist&utm_campaign=1_1) | Give unadjusted estimates and, if applicable, confounder-adjusted estimates and their precision (e.g., 95% confidence intervals). Make clear which confounders were adjusted for and why they were included. | Abstract – Results; Results – 3.2 and 3.3; Table 2 (unadjusted and adjusted regression coefficients with 95% CIs; adjustment for caregiver age, sex, daily caregiving hours, and LTCI care level). |
| [16b. Main results – category boundaries](https:/resources.equator-network.org/reporting-guidelines/strobe/items/main-results-category-boundaries.html?utm_source=strobe&utm_medium=checklist&utm_campaign=1_1) | Report category boundaries when continuous variables were categorised. | Materials and methods – 2.2 Survey method (LTCI care level categories: support 1–2 and nursing care 1–5); Table 1 (distribution of care levels). No categorisation of SOC scores, caregiving hours, or VAS outcome in regression analyses. |
| [16c. Main results – risk](https:/resources.equator-network.org/reporting-guidelines/strobe/items/main-results-risk.html?utm_source=strobe&utm_medium=checklist&utm_campaign=1_1) | If relevant, consider translating estimates of relative risk into absolute risk for a meaningful time period. | Not applicable – caregiver burden was analysed as a continuous VAS score; no relative risk estimates were produced or translated into absolute risk. |
| [17. Other analyses](https:/resources.equator-network.org/reporting-guidelines/strobe/items/other-analyses.html?utm_source=strobe&utm_medium=checklist&utm_campaign=1_1) | Report other analyses done—e.g., analyses of subgroups and interactions, and sensitivity analyses. | Discussion – 3.4.2 Association between family caregiver burden and SOC (reference to additional correlation analyses); Supplementary File 2, Table S1 (correlation matrix among study variables). No subgroup or sensitivity analyses were performed. |
| **Discussion** |  |  |
| [18. Key results](https:/resources.equator-network.org/reporting-guidelines/strobe/items/key-results.html?utm_source=strobe&utm_medium=checklist&utm_campaign=1_1) | Summarise key results with reference to study objectives. | Discussion – 3.4 and 3.4.2; Conclusion section (summary of key findings in relation to the study objectives). |
| [19. Limitations](https:/resources.equator-network.org/reporting-guidelines/strobe/items/limitations.html?utm_source=strobe&utm_medium=checklist&utm_campaign=1_1) | Discuss limitations of the study, taking into account sources of potential bias or imprecision. Discuss both direction and magnitude of any potential bias. | Discussion – 3.4.3 Significance and Limitations of the Study (cross-sectional design and lack of causal inference, limited covariates and residual confounding, possible selection bias from complete-case analysis, and small sample size). |
| [20. Interpretation](https:/resources.equator-network.org/reporting-guidelines/strobe/items/interpretation.html?utm_source=strobe&utm_medium=checklist&utm_campaign=1_1) | Give a cautious overall interpretation considering objectives, limitations, multiplicity of analyses, results from similar studies, and other relevant evidence. | Discussion – 3.4.2 and 3.4.3; Conclusion section (overall interpretation of the association between SOC and caregiver burden in light of previous research and study limitations). |
| [21. Generalisability](https:/resources.equator-network.org/reporting-guidelines/strobe/items/generalisability.html?utm_source=strobe&utm_medium=checklist&utm_campaign=1_1) | Discuss the generalisability (external validity) of the study results. | Discussion – 3.4.2 and 3.4.3 (generalisability to family caregivers of older adults using day-care and day-care rehabilitation services under Japan’s LTCI system; limited generalisability to other caregiving contexts). |
| **Other information** |  |  |
| [22. Funding](https:/resources.equator-network.org/reporting-guidelines/strobe/items/funding.html?utm_source=strobe&utm_medium=checklist&utm_campaign=1_1) | Give the source of funding and the role of the funders for the present study and, if applicable, for the original study on which the present article is based. | Funding statement ("This research did not receive any specific grant from funding agencies in the public, commercial, or not-for-profit sectors."). |

## 1 How to specify where content is

Tell the reader where they can find information. E.g.,

- Results; paragraph 2
- Methods, Participants; paragraphs 1 & 2.
- Table 3
- Supplement B, para. 4

If you have chosen not to describe an item, explain why. You can do this in the checklist, or as a note below it.

You can describe items in the article body, or in tables, figures, or supplementary materials, and should prioritize items you feel are most important to your intended audience. The order of items in your manuscript does not need to match the order of items in this checklist. You can decide how best to structure your work.

## 2 How to cite

Describe how you used STROBE at the end of your Methods section, referencing the resources you used e.g.,

‘We used the STROBE reporting guideline(1) to draft this manuscript, and the STROBE reporting checklist(2) when editing, included in supplement A’

If you use a reporting checklist, remember to include it as a supplement when publishing so that readers can easily find information and see how you have interpreted the guidance.

1. Elm E von, Altman DG, Egger M, Pocock SJ, Gøtzsche PC, Vandenbroucke JP, et al. The strengthening the reporting of observational studies in epidemiology (STROBE) statement: Guidelines for reporting observational studies. Annals of Internal Medicine [Internet]. 2007 Oct;147(8):573–7. Available from: <https://www.acpjournals.org/doi/10.7326/0003-4819-147-8-200710160-00010>

2. Elm E von, Altman DG, Egger M, Pocock SJ, Gøtzsche PC, Vandenbroucke JP, et al. The STROBE reporting checklist. In: Harwood J, Albury C, Beyer J de, Schlüssel M, Collins G, editors. The EQUATOR network reporting guideline platform [Internet]. The UK EQUATOR Centre; 2025. Available from: [https:/resources.equator-network.org/reporting-guidelines/strobe/strobe-checklist.docx](https://https:/resources.equator-network.org/reporting-guidelines/strobe/strobe-checklist.docx)
