# Supplementary material for: Relationship Between Caregiver Burden and Sense of Coherence in Home‐Based Family Caregivers
Source: Occup Ther Int. 2026 Jan 28;2026:6638411. doi: 10.1155/oti/6638411 (PMC12848607; doi:10.1155/oti/6638411)
Supplement: Supplementary file 2 — Supporting Information 2 Table S1: Correlation matrix among study variables. [file OTI-2026-6638411-s002.docx]

# Supplementary Table S1. Correlation matrix among study variables

| Variable | 1 | 2 | 3 | 4 | 5 | 6 | 7 | 8 |
| --- | --- | --- | --- | --- | --- | --- | --- | --- |
| 1. Caregiver Burden | 1.00 | -.535** | -.501** | -.382** | -.099 | -.033 | .389** | .330** |
| 2. Comprehensibility | -.535** | 1.00 | .829** | .642** | .090 | -.101 | -.057 | -.020 |
| 3. Manageability | -.501** | .829** | 1.00 | .709** | .199 | -.090 | -.037 | -.104 |
| 4. Meaningfulness | -.382** | .642** | .709** | 1.00 | .115 | .077 | -.052 | -.149 |
| 5. Caregiver Age | -.099 | .090 | .199 | .115 | 1.00 | .094 | .396** | -.024 |
| 6. Caregiver Sex | -.033 | -.101 | -.090 | .077 | .094 | 1.00 | .182 | .005 |
| 7. Daily Caregiving Hours | .389** | -.057 | -.037 | -.052 | .396** | .182 | 1.00 | .286* |
| 8. LTCI Level | .330** | -.020 | -.104 | -.149 | -.024 | .005 | .286* | 1.00 |

Note. Values are Pearson correlation coefficients (r). *p < .05, **p < .01.
